# Transepithelial Secretion of Mucosal IgM Mediated by Polymeric Immunoglobulin Receptor of Flounder (*Paralichthys olivaceus*): *In-Vivo* and *In-Vitro* Evidence

**DOI:** 10.3389/fimmu.2022.868753

**Published:** 2022-04-06

**Authors:** Xiuzhen Sheng, Yuan Guo, Hui Zhu, Baihui Chai, Xiaoqian Tang, Jing Xing, Heng Chi, Wenbin Zhan

**Affiliations:** ^1^ Laboratory of Pathology and Immunology of Aquatic Animals, Key Laboratory of Mariculture of Ministry of Education (KLMME), Ocean University of China, Qingdao, China; ^2^ Laboratory for Marine Fisheries Science and Food Production Processes, Qingdao National Laboratory for Marine Science and Technology, Qingdao, China

**Keywords:** polymeric immunoglobulin receptor (pIgR), secretory IgM (SIgM), transepithelial secretion, Madin–Darby canine kidney (MDCK), transcytosis, flounder (*Paralichthys olivaceus*)

## Abstract

Secretory immunoglobulin (SIg) is crucial for mucosal surface defenses, but the transepithelial secretion of SIg mediated by polymeric immunoglobulin receptor (pIgR) is not clarified in fish. We previously found that flounder (*Paralichthys olivaceus*) pIgR (fpIgR) and secretory IgM (SIgM) increased in gut mucus post-vaccination. Here, the fpIgR-positive signal was mainly observed in the intestinal epithelium, whereas the IgM-positive signal was mainly distributed in the lamina propria, before immunization. IgM signals increased in the lamina propria and then in the epithelium after immunization with inactivated *Vibrio anguillarum*, and co-localization between IgM and fpIgR in the epithelium was determined, while the presence of EdU^+^IgM^+^ cells in the lamina propria identified the proliferative B cells, revealing that the secretion and transepithelial transport of SIgM locally occurred in the gut of flounder. Subsequently, we established an *in-vitro* model of transfected MDCK cells that stably expressed the fpIgR. After a recombinant eukaryotic expression plasmid (pCIneoEGFP-fpIgR) was constructed and transfected into MDCK cells, stable expression of the fpIgR in transfected MDCK-fpIgR cells was confirmed, and the tightness and integrity of the polarized cell monolayers grown on Transwells were evaluated. Afterward, the serum IgM of flounder was purified as a binding ligand and placed in the lower compartment of Transwells. An ~800-kDa protein band in the upper compartment was shown to be IgM- and fpIgR-positive, and IgM-positive fluorescence was seen in MDCK-fpIgR cells but not in MDCK-mock cells. Hence, the fpIgR helped polymeric IgM to pass across MDCK-fpIgR cells *via* transcytosis in a basolateral-to-apical fashion. These new findings provide a better understanding of the pathways shaping mucosal IgM responses and the local mucosal immune mechanisms in teleosts.

## 1 Introduction

Secretory immunoglobulin (SIg) is very important for mucosal surface defenses. SIg is produced by plasma cells in the lamina propria and transported across glandular and epithelial cells into mucosal secretions to exert protective functions (1, 2). This process (referred to as “transcytosis”) is inseparable from the polymeric immunoglobulin receptor (pIgR), a type I transmembrane glycoprotein and member of the Ig superfamily (3). The mammalian pIgR is expressed by the epithelial cells of skin-, gastrointestinal tract-, and respiratory tract-associated lymphoid tissues and glandular epithelial cells of the liver and breast, and it is responsible for transporting polymeric Ig (dimeric IgA and, in some mammals, pentameric IgM) to exocrine secretions (4, 5). In mammals, transcytosis from the basolateral surface of the epithelium to the apical side has been studied in cell models *in vitro*, such as Madin–Darby canine kidney (MDCK) cells (a commonly used epithelial model system) (6, 7). The locally synthesized polymeric immunoglobulins (pIgs) bind to the pIgR anchored on the basolateral side of epithelial cells, and after receptor-mediated endocytosis, the pIg-bound or unbound pIgR is transported through a series of intracellular vesicles. Upon reaching the luminal side, the extracellular region of the pIgR [called the secretory component (SC)] is cleaved proteolytically to release the SC–pIg complex, thereby forming SIg (4, 5, 8). However, how the mucosal immune system defends teleosts (ray-finned fishes) is far from clear as compared with that in mammals; hence, more knowledge on the mucosal immunity of teleosts must be generated (9).

In teleosts, tetrameric IgM and dimeric IgT (but not dimeric IgA) have been identified in the gut, skin, and gill mucus (10, 11). Studies indicate that IgM is the principal Ig involved in humoral and mucosal immunity in bony fish, whereas IgT (found only in teleosts) is thought to be a specialized mucosal Ig and acts as an equivalent of the phylogenetically distant mammalian IgA (11–13). Teleost fish express the oldest recognizable pIgR protein. To date, the pIgR in many fish species has been cloned and analyzed and confirmed to be expressed predominantly in the skin, gills, intestine, and liver. pIgR levels fluctuate slightly or drastically in different tissues at different times after fish are challenged with the Gram-negative bacteria *Aeromonas hydrophila* (14) and *Vibrio anguillarum* (15) or the Gram-positive bacteria *Streptococcus iniae* and snakehead rhabdovirus (16), suggesting the potential function of the pIgR in vaccination strategies. The pIgR expressed in fish skin highlights the importance of SIg as a mucosal barrier against pathogens in an aquatic environment compared with no expression of the pIgR in mammalian skin (17). In addition, IgM and the pIgR found in the bile and liver of teleosts demonstrate the possible existence of hepatobiliary transport in teleosts, which can modulate intestinal immunity to some extent (15, 18). Fewer studies on the function of the pIgR in teleosts have been undertaken as compared with those in mammals. Nevertheless, the pIgR has been shown to bind to IgM in fugu (*Takifugu rubripes*), carp (*Cyprinus carpio*), orange-spotted grouper (*Epinephelus coioides*), and flounder (*Paralichthys olivaceus*) (17, 19–21) or to IgT in rainbow trout (*Salmo gairdneri*) and dojo loach (*Misgurnus anguillicaudatus*) (13, 22) in mucus (but not in serum). Flounder pIgR is found to mediate polymeric IgM–antigen complexes across the intestinal epithelium into gut mucus (23). In the flounder gill (FG) cell model, pIgR expression and SC production are upregulated by cytokine tumor necrosis factor-α *via* activating the PI3K and NF-κB signaling pathways (24). However, the mechanism by which mucosal Igs gain access to the lumen across mucosal epithelium remains undefined, and direct lines of evidence for pIgR-mediated transcytosis of pIgs have not been shown in teleost fish. Hence, comprehensive studies on pIgR function and SIg transcytosis in teleosts can aid our understanding of mucosal immune defense systems (9, 25).

Previously, we cloned the flounder pIgR (fpIgR) and developed mouse anti-fpIgR polyclonal antibodies (21). In the present study, after flounders were immunized with inactivated *V. anguillarum*, *in-vivo* evidence for transepithelial transport of fpIgR–IgM complexes in gut-associated lymphoid tissue was sought by double-immunofluorescence staining and confocal laser scanning microscopy (CLSM) and measured quantitatively by ImageJ software at different times. Also, proliferative IgM^+^ B cells were identified by injection of 5-ethynyl-2′-deoxyuridine (EdU). Due to the lack of fpIgR-deficient fish, to verify that transepithelial transport of SIgM was mediated by the fpIgR, an *in-vitro* MDCK-fpIgR cell model was established and confirmed by reverse transcription-polymerase chain reaction (RT-PCR), Western blotting, and indirect immunofluorescence. The tightness and integrity of MDCK-fpIgR cell monolayers were evaluated by measuring their transepithelial electrical resistance (TEER), the apparent permeability coefficients (Papp) of Lucifer yellow, and expression of the tight-junction protein ZO-1. Western blotting under native polyacrylamide gel electrophoresis (native-PAGE) conditions was done to show that the fpIgR could transport tetrameric IgM, and IgM location in MDCK-fpIgR cells was observed by CLSM.

## 2 Materials and Methods

### 2.1 Ethical Approval Statement

This study was conducted in strict accordance with the recommendations in the Guide for the Use of Experimental Animals of the Ocean University of China. The protocols for animal care and handling were approved by the Animal Care and Use Committee of the Ocean University of China (Permit Number: 20180101). All efforts were made to minimize the suffering of fish.

### 2.2 Fish Immunization and Tissue Sampling

A total of 150 healthy flounders (length, 15–17 cm) were obtained from a fish farm in Rizhao, Shandong Province of China. Flounders were maintained in aerated running seawater at 21°C ± 1°C and fed a commercial diet twice daily. After acclimatization to the laboratory setting for 7 days, flounders were divided randomly into two groups (three replicates in each group) for immunization.

Formalin-killed *V. anguillarum* was prepared, and the safety of the inactivated bacteria was checked. The concentration of inactivated bacterin was adjusted to 1 × 10^8^ CFU/ml in phosphate-buffered saline (PBS), and fish were intraperitoneally injected with 100 µl of inactivated bacterin or an equal volume of sterile PBS as control as described previously (15). Before sacrificing and handling, fish were anesthetized with ethyl 3-aminobenzoate methanesulfonic acid (MS222, Sigma, USA) to minimize suffering. Nine fish in each group were sampled randomly for collection of the hindgut before immunization (0 day) as well as at 3, 7, 14, 21, and 28 days post-immunization (dpi). Hindguts were immersed in a tissue-freezing medium (optimum cutting temperature compound, OCT; Leica, Wetzlar, Germany) immediately and frozen at −80°C. Subsequently, 5-µm-thick sections were cut and fixed with cold acetone for 10 min and, after air-drying in a fume cupboard, stored at −20°C before use.

To confirm the proliferation of IgM^+^ B cells in the hindgut, on the seventh day after intraperitoneal injection of inactivated *V. anguillarum*, EdU (EdU powder dissolved with PBS, 1.5 mg per individual, CellorLab, China) was intraperitoneally injected to immunized and control fish. At 48 h after EdU injection, the hindguts were collected from nine fish in each group and used for cryostat section preparation.

### 2.3 Double-Immunofluorescence Labeling and Co-Localization in Hindgut *In Vivo*

To determine the co-localization between IgM and the fpIgR in the intestinal mucosa of flounders after *V. anguillarum* immunization, cryostat sections of hindguts were used for double staining of IgM and the fpIgR. Cryostat sections were washed in PBS containing 0.1% Tween-20 (PBST) for 10 min. Following incubation with 5% bovine serum albumin (BSA) at room temperature for 1 h to block the binding of non-specific antibodies, the sections were incubated with mixtures (1:1, *v*/*v*) of mouse anti-IgM monoclonal antibody (MAb) 2D8 (1:1,000 dilution) (26) and rabbit anti-fpIgR antibody (1:1,000) (21) for 1 h at 37°C. After three washes with PBST, the sections were incubated with a mixture (1:1, *v*/*v*) of Alexa 488-conjugated goat anti-mouse IgG (1:1,000; Thermo Scientific, Waltham, MA, USA) and Cy3-conjugated goat anti-rabbit IgG (1:400; Sigma-Aldrich, Saint Louis, MO, USA) for 1 h at 37°C. Thereafter, redundant antibodies were washed off, and sections were stained with 4′,6-diamidino-2-phenylindole (DAPI; 1:1,000; Thermo Scientific) for 10 min at room temperature to enable imaging by CLSM and nuclei visualization. Quantitative analyses of co-localization between IgM and the fpIgR in intestinal mucosa were done using ImageJ image analysis software (National Institutes of Health, Bethesda, MD, USA).

Furthermore, cryostat sections of the hindgut from EdU-injected fish were utilized for IgM and EdU co-staining. In brief, after washing in PBST for 10 min, the sections were incubated with 0.5% Triton X-100 in PBS for 20 min and then blocked with 5% BSA for 40 min at 37°C. Following three washes with PBST, the sections were incubated with EdU solution (CellorLab, China) containing Alexa Fluor 649 azide for 30 min in the dark. Then, the sections were incubated with mouse anti-flounder IgM MAb (1:1,000) as primary antibody and Alexa Fluor 488 conjugated goat anti-mouse IgG (1:1,000, Sigma, USA) as secondary antibody. After washing off the redundant antibodies, the sections were stained with DAPI (1:1,000, Sigma, USA) for 10 min to visualize the nucleus and observed by Zeiss Imager Z2 fluorescence microscope.

### 2.4 Construction of Eukaryotic Plasmid Containing the fpIgR Gene

The full-length open-reading frame of the fpIgR was amplified by PCR using the recombinant plasmid (pET-32a-fpIgR) constructed previously by our research team (21) as a template. The specific forward primer (CCGGAATTCGCCACCATGGCGCAACTCTTCACACTCA) and reverse primer (GGCGTCGACTTGTGCATAAAAACATCCTGGGAATCC) containing *Eco*RI and *Sal*I restriction sites (underlined) were designed according to the published sequence of the fpIgR (HM536144.1) and multiple cloning site sequence of eukaryotic vector pCIneoEGFP (Addgene, Cambridge, MA, USA). The PCR product was analyzed by electrophoresis on 1% agarose gel and purified with a DNA Gel Extraction kit (Tiangen Biotech, Tiangen, China). The fpIgR amplicon was ligated into plasmid pCIneoEGFP after digestion with *Eco*RI and *Not*I, and then the recombinant plasmid (pCIneoEGFP-fpIgR) was transformed into *Escherichia coli* DH5α cells (TransGen Biotech, Beijing, China). Positive clones were screened and then confirmed by sequencing. The sterile eukaryotic vectors pCIneoEGFP and pCIneoEGFP-fpIgR used in the subsequent transfection were extracted using an EndoFree Plasmid kit (Tiangen Biotech), and the recombinant plasmid was verified by double digestion.

### 2.5 Transfection and Screening of Monoclonal MDCK Cell Line Expressing the fpIgR

The MDCK strain II (CRL-2936; American Type Culture Collection, Manassas, VA, USA) was cultured in minimum essential medium (MEM; Gibco, Billings, MT, USA) supplemented with 10% fetal bovine serum (FBS; Gibco) at 37°C in an atmosphere of 5% CO_2_. Cells were subcultured 1 day in advance to ensure that, on the day of transfection, those cells at ~90% confluence were trypsinized. Then, the cell density was adjusted to ~2 × 10^6^ cells per microcentrifuge tube. To prepare the transfection complex for one tube of cells, 16 μl of Lipofectamine 3000 (Invitrogen, Carlsbad, CA, USA) diluted in 125 μl of Reduced Serum Medium (Opti-MEM; Gibco) and 4 μg of plasmid DNA together with 8 μl of P3000 diluted in 125 μl Opti-MEM were mixed gently and incubated for 15 min at room temperature. Cell pellets were collected by centrifugation at 1,000×*g* for 5 min at room temperature and then resuspended in 100 μl of the complex. After incubation (37°C, 5% CO_2_) for 20 min, the cells in each microcentrifuge tube with another 2 ml of Opti-MEM were seeded in one well of a six-well plate. Following incubation for 6 h, the medium was aspirated and replaced with 2 ml of fresh growth medium to culture cells for another 24 h. Transfected cells (1:20 dilution) and untransfected cells (control) were seeded on new six-well plates with a selection medium (MEM containing 600 μg/ml of G418 and 10% FBS). The selection period lasted for 2 weeks, with the medium replaced every 3 days until there were no untransfected cells. Surviving cells expressing enhanced green fluorescent protein (EGFP), as verified by inverted fluorescence microscopy, were purified according to the limited dilution method. Briefly, positive colonies were diluted to ~10 cells/ml in the selection medium and seeded on several 96-well plates. During this period, the selection medium was changed every 3 days, and the wells with only one cell were marked and observed until a monoclonal cell line with stable expression of EGFP or the fpIgR, respectively, was obtained. fpIgR expression in MDCK-fpIgR cells and EGFP expression in MDCK-mock cells were confirmed by RT-PCR, Western blotting, and immunofluorescence microscopy.

### 2.6 RT-PCR

Total RNA was extracted from MDCK, MDCK-mock, and MDCK-fpIgR cells using TRIzol reagent (TaKaRa Biotechnology, Shiga, Japan). Each sample was dissolved in 1 ml of TRIzol reagent by homogenization according to the manufacturer’s instructions. RNA (1 μg) was converted to cDNA using a PrimeScript RT Reagent kit with gDNA Eraser (TaKaRa Biotechnology). cDNA samples were used for subsequent PCR amplification to measure the expression of EGFP and the fpIgR. Amplifications were carried out using two pairs of primers. One pair was EGFP forward (ATGGTGAGCAAGGGCGAGG) and EGFP reverse (TTACTTGTACAGCTCGTCCATGCC) with an expected PCR product of 720 bp. The other pair was fpIgR forward (ATGGCGCAACTCTTCACACTCAC) and fpIgR reverse (TCAGTGCATAAAAACATCCTGGGAA) with an expected PCR product of 1,005 bp. PCR products were analyzed by electrophoresis on 1% agarose gel.

### 2.7 Western Blotting

To determine the subcellular distribution of the fpIgR, MDCK cells and MDCK-fpIgR cells stably expressing the fpIgR, and MDCK-mock cells expressing EGFP, were broken by repeated freezing and thawing and then fractionated into the cell membrane and cytoplasmic portions using a Membrane and Cytosol Protein Extraction kit (Beyotime, Beijing, China) according to the manufacturer’s instructions. Subcellular proteins were subjected to 10% sodium dodecyl sulfate-polyacrylamide gel electrophoresis (SDS-PAGE) and transferred onto polyvinylidene fluoride (PVDF) membranes (Millipore, Bedford, MA, USA). Initially, the PVDF membranes were blocked with 5% BSA in PBS for 1 h at 37°C and then washed thrice with PBST for 5 min between each subsequent step. Mouse anti-GFP tag MAb (Abbkine, Beijing, China) diluted 1:1,000 in PBS and goat anti-mouse IgG antibody conjugated with alkaline phosphatase (AP) (Millipore) diluted 1:5,000 in PBS were used successively to incubate PVDF membranes for 1 h at 37°C.

To further determine the ability of MDCK-fpIgR cells to produce the SC of the fpIgR, the culture medium without FBS was collected from the cell culture flasks and concentrated by ultrafiltration with an exclusion limit of 10 kDa (Millipore). The supernatants were subjected to Western blotting under reducing conditions as described above. Mouse anti-fpIgR polyclonal antibody (21) diluted 1:2,000 in PBS and AP-labeled goat anti-mouse IgG (Millipore) diluted 1:5,000 in PBS were used as the primary and secondary antibodies, respectively. As a negative control, MDCK-mock cell culture supernatants without FBS were used. Moreover, the gut mucus sampled from the immunized flounder previously (15) and culture supernatants of FG cells without FBS served as positive controls.

### 2.8 Immunofluorescence Microscopy

The MDCK-mock and MDCK-fpIgR cells grown on round coverslips in 24-well plates for 24 h were washed thrice with ice-cold PBS (5 min each time). After incubation with 4% paraformaldehyde for 30 min at room temperature, the cells were washed thrice with PBS for a total of 15 min and then blocked in PBS containing 5% BSA for 1 h at 37°C. This was followed by washing (as described above) and incubation overnight at 4°C with a mouse anti-fpIgR polyclonal antibody diluted 1:1,000 in PBS. The next day, the cells were washed thrice with PBST for 15 min and incubated for 45 min at 37°C with Cy3-labeled anti-mouse IgG as the secondary antibody (Invitrogen). Cell nuclei were visualized by DAPI staining for 10 min at room temperature. Finally, the excised cell coverslips were mounted on glass slides using an antifade mounting medium (Solarbio, Beijing, China) and observed under a fluorescence microscope (Zeiss, Germany).

### 2.9 Establishment of a Polarized Monolayer of MDCK-fpIgR Cells

#### 2.9.1 Localization of the fpIgR in the Polarized Monolayer of Cells

To generate polarized cell monolayers in Transwells, MDCK-mock and MDCK-fpIgR cells in the logarithmic growth phase were seeded at ~5 × 10^4^ cells/filter onto 6.5-mm Transwell polyester filters (Corning Costar, Corning, NY, USA). The upper and lower compartments of Transwells were filled with 0.2 and 1 ml of medium, respectively. The medium was replaced every 2 days, and the morphology and growth of the cells were observed daily under an inverted microscope.

To ascertain if the cells on the Transwell semipermeable membrane were a polarized monolayer and the location of fpIgR expression in MDCK-fpIgR cells, the cells grown on Transwell semipermeable membrane were imaged using CLSM. The fluorescence images of each layer were superimposed, and three-dimensional (3D) volumes of the *z*-axis (spacing of 1 μm between single confocal slices) were reconstructed. These micrographs were representative slices of all the fluorescence images, and localization of the fpIgR was assessed in 3D in whole-cell volumes.

#### 2.9.2 TEER Measurement and Penetration by Fluorescein Sodium

TEER was measured with the Millicell ERS-2 Voltohmmeter (Millipore) every day. TEER of different sites in each chamber was measured at least thrice and averaged, and the experiment was repeated three times.

The absorbance of a standard solution of fluorescein sodium (Sigma-Aldrich) with gradient concentrations of 5, 2.5, 1.25, 0.625, 0.313, and 0.156 μg/ml at an absorption wavelength of 460 nm was detected by a microplate reader so that a standard curve could be drawn. Cell monolayers cultured for 4 days were washed twice with Hank’s balanced salt solution (HBSS) preheated to 37°C for 5 min each time. Then, 0.2 ml of fluorescein sodium solution (330 μg/ml) was added to the apical chamber of Transwells, whereas 1 ml of HBSS solution was added into the basolateral chamber. The incubation lasted for 30, 60, 90, and 120 min at 37°C in an atmosphere of 5% CO_2_, respectively. The absorbance of different samples was measured with a microplate reader to calculate the Papp of fluorescein sodium in each chamber based on the standard curve. This experiment was repeated three times.

#### 2.9.3 Immunofluorescence Staining of ZO-1

Polarized monolayers of MDCK-mock and MDCK-fpIgR cells were grown on 6.5-mm filters for 3–4 days. The filters were washed thrice in ice-cold PBS (5 min each time) and then incubated on both sides of the chamber with 4% paraformaldehyde for 20 min. Cells were washed thrice with PBS and blocked in PBS containing 5% BSA for 1 h at 37°C and washed as described above. Following incubation overnight at 4°C with a rabbit anti-ZO-1 polyclonal antibody (Proteintech, Chicago, IL, USA) diluted 1:400 in PBS, cells were washed thrice with PBST for 15 min and incubated with Cy3-labeled anti-rabbit IgG antibody for 45 min at 37°C. After DAPI staining for intact nuclei, the polyester membrane was cut down with a scalpel and mounted on glass slides using the antifade mounting medium before observation by CLSM.

### 2.10 Detection of fpIgR-Mediated IgM Transcytosis in MDCK-fpIgR Cells

#### 2.10.1 Isolation of Serum IgM of Flounders

The serum IgM of flounders was purified as described previously (26) with some modification. Briefly, the Ig of flounders was first purified from serum by precipitation with 50% saturated ammonium sulfate. The precipitate was pelleted by centrifugation at 14,000×*g* for 30 min at 4°C, resuspended in 50 mM PBS (pH 7.2), and dialyzed extensively against the same buffer. The dialyzed solution was fractionated further by a HiPrep16/60 Sephacryl S-300 HR gel-filtration column (GE Healthcare, Piscataway, NJ, USA) using a protein purification system (AKTA prime; Amersham, UK). After verification by SDS-PAGE and Western blotting under native-PAGE conditions, the protein sample was concentrated in HBSS buffer by ultrafiltration at a limit of 100 kDa (Millipore).

#### 2.10.2 Detection of fpIgR (SC)–IgM Complexes Transported by the Cell Model

An fpIgR-mediated transcytosis assay in polarized MDCK-fpIgR cells was carried out as described previously (27, 28) with slight modification. Polarized monolayers of MDCK-mock and MDCK-fpIgR cells were grown on 6.5-mm filters for 3–4 days. Then, the filters were washed thrice in HBSS at 37°C. Next, 200 μl of HBSS was added to the apical chamber of Transwells, and the filter was placed onto a 500-μl drop of HBSS with 0.1 mg/ml of the serum IgM of flounders for 1 and 2 h, respectively. At each time point, the medium in the apical chamber was collected immediately and analyzed by Western blotting under native-PAGE conditions. Samples were subjected to gradient separation using 3% stacking gels and 3%–20% gradient gels in the absence of SDS, then electrophoresis was conducted for 19 h at 20 mA at 4°C. After the samples had been transferred onto PVDF membranes (Millipore), they were blocked with 5% BSA in PBS for 1 h at 37°C. After washing thrice with PBST, the PVDF membranes were incubated with anti-serum IgM MAb 2D8 (26) and anti-fpIgR polyclonal antibody diluted 1:1,000 in PBS for 1 h at 37°C, respectively. After washing again, the PVDF membranes were incubated with goat anti-mouse IgG antibody conjugated with AP diluted 1:5,000 in PBS for 1 h at 37°C.

#### 2.10.3 IgM Detection in MDCK-fpIgR Cells

After the samples in the apical chamber of Transwells were collected, MDCK-fpIgR cells and MDCK-mock cells were washed thrice and then incubated with anti-serum IgM MAb 2D8 (1:1,000) for 1 h at 37°C. After redundant antibodies had been washed off, the cells were incubated with Alexa 647-conjugated goat anti-mouse IgG (1:1,000) for 45 min at 37°C and washed again. Subsequently, the cells on the membrane were stained with DAPI (1:1,000) for 10 min at room temperature to enable detection by CLSM and nuclei visualization. MDCK-fpIgR cells incubated with non-immune mouse serum and MDCK-mock cells incubated with anti-serum IgM MAb 2D8 served as negative controls. Transcytosed IgM in MDCK-fpIgR cells was checked by CLSM z-scan.

### 2.11 Statistical Analyses

Statistical analyses were carried out using SPSS v22.0 (IBM, Armonk, NY, USA). Data were presented as the mean ± SEM. Differences were analyzed by one-way analysis of variance (ANOVA) and Dunn’s multiple comparison tests. *p <*0.05 was considered significant.

## 3 Results

### 3.1 *In-Vivo* Evidence for Transepithelial Transport of fpIgR–IgM Complexes

**Figure 1:**
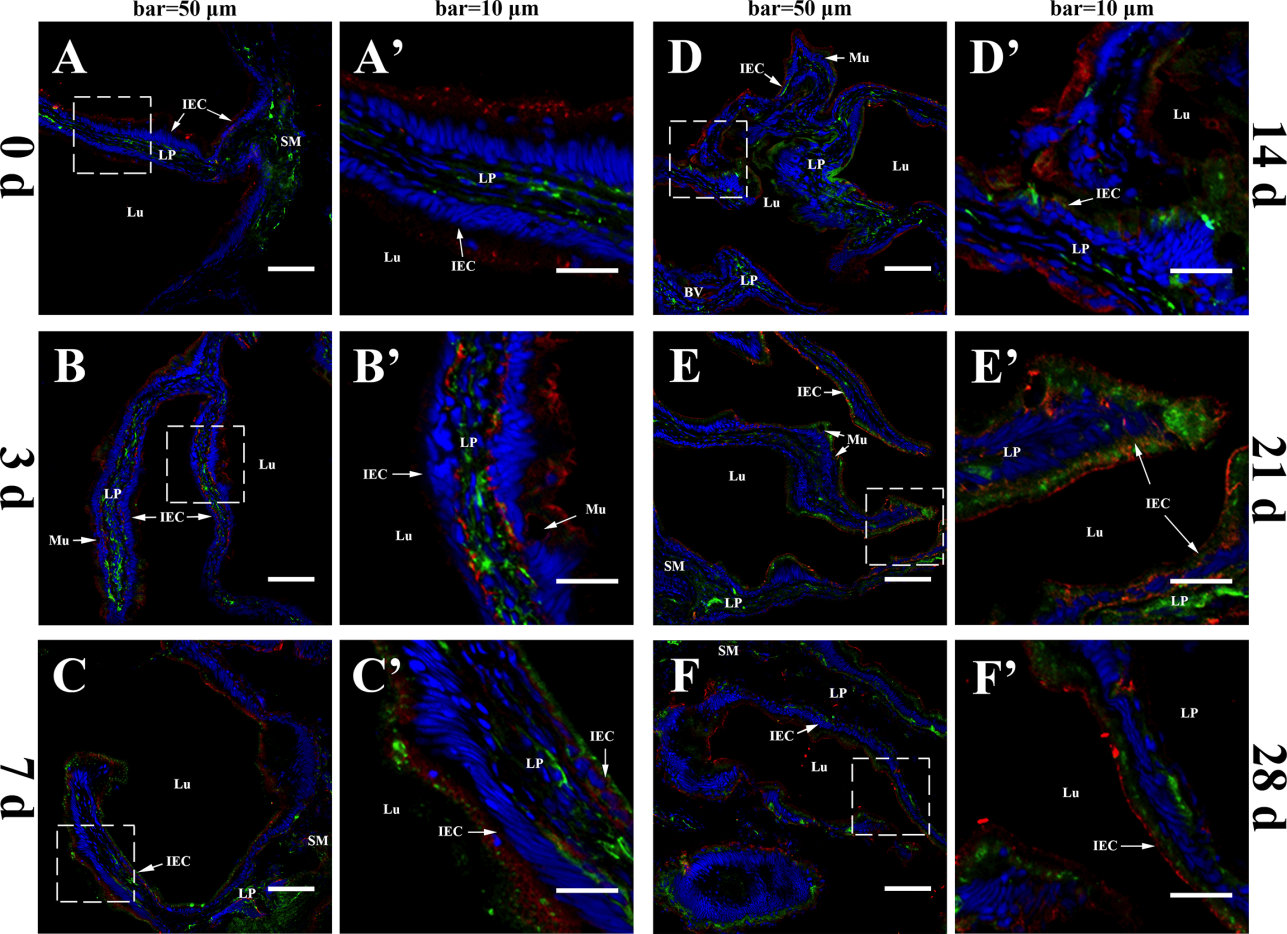
*In-vivo* evidence of transepithelial transport of the fpIgR–IgM complex in flounder. The IgM and the fpIgR in the hindgut were double-stained at different times after the flounder was immunized with inactivated *Vibrio anguillarum*. **(A–F)** Observation of co-localization of IgM and the fpIgR was at 0, 3, 7, 14, 21, and 28 days post-immunization (dpi), respectively. IgM-positive green fluorescence was located in the lamina propria at 0 dpi and then presented and increased in intestinal epithelial cells from 3 to 28 dpi, whereas fpIgR-positive red fluorescence was mainly in epithelial cells. Bar = 50 µm. **(A′–F′)** A higher magnification view of the insert area (white box) in **(A–F)**, respectively. Bar = 10 µm. Cell nuclei were counterstained in blue with DAPI. LP, lamina propria; Lu, lumen; IECs, intestinal epithelial cells; Mu, mucus cells; BV, blood vessel; SM, submucosa.

The merged images of intestinal mucosa double-stained for IgM and fpIgR are shown in **Figures 1A–F**, Before immunization, IgM-positive green fluorescence was distributed mainly in the lamina propria of the gut, whereas fpIgR-positive red fluorescence was located mainly in intestinal epithelial cells (**Figures 1A, A'**). After inactivated *V. anguillarium* immunization, the intensity of IgM-positive signals increased in the lamina propria and then in the intestinal epithelium over time, and the intensity of fpIgR-positive signals was also boosted (**Figures 1B–F**). Specifically, at 3 dpi, green fluorescence for IgM in the lamina propria was strengthened, the red signals for the fpIgR presented at the base of the cell, and some yellow fluorescence signals (which might represent co-localization of IgM and the fpIgR) were observed at epithelial cell bases (**Figures 1B, B**'). At 7 dpi, IgM-positive green fluorescence was observed in intestinal epithelial cells (**Figures 1C, C'**), which was enhanced obviously at 14 and 21 dpi (**Figures 1D, E'**) and then weakened slightly at 28 dpi (**Figures 1F, F′**).

**Figure 2:**
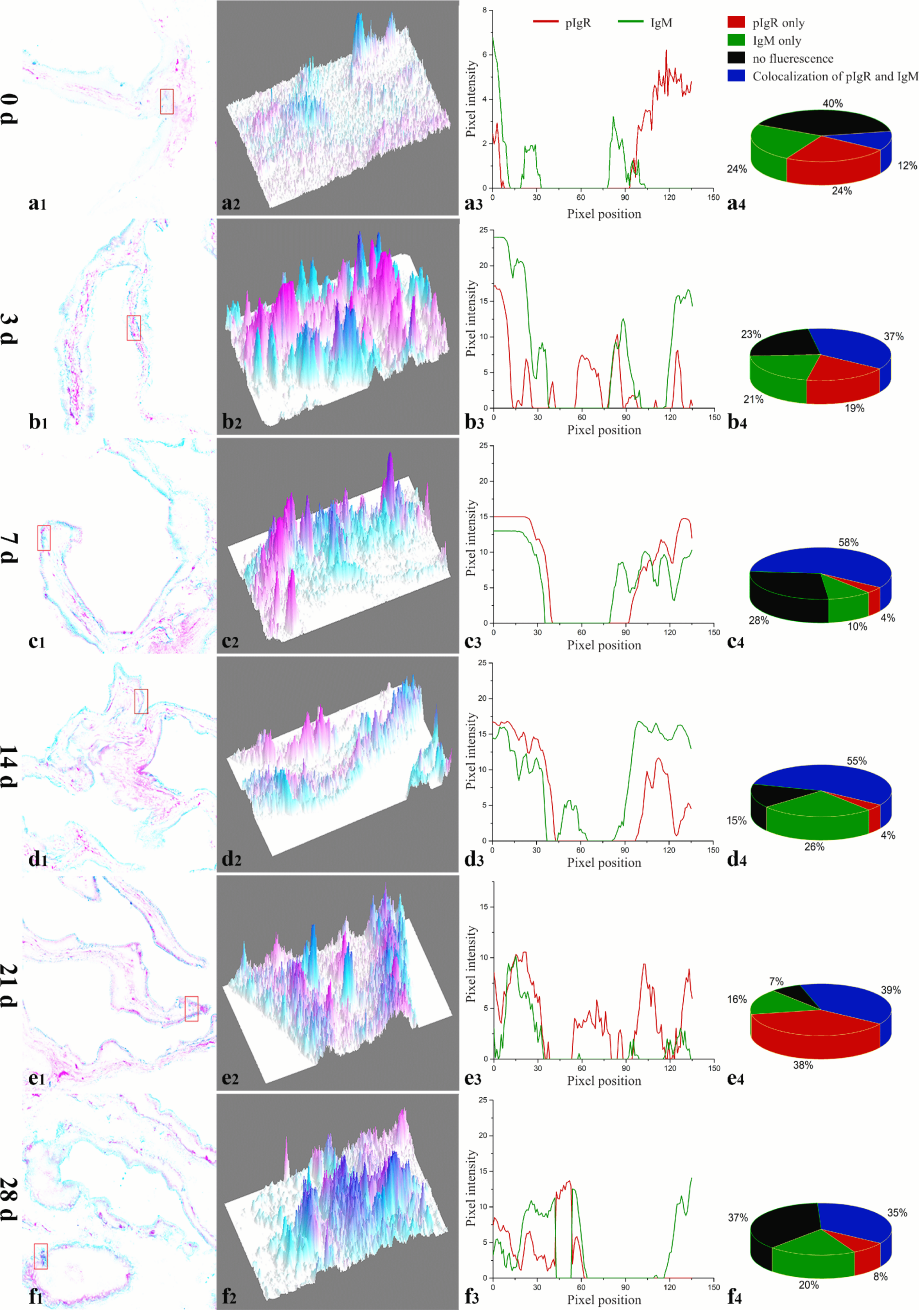
Quantitative image analyses for co-localization of IgM and the fpIgR in a selected area of the intestinal epithelium in **Figures 1A–F** using ImageJ at different times after immunization. **(a1–f1)** A new merged image of **Figures 1A–F** using green and red to represent the positive signals of the fpIgR and IgM in the intestinal epithelium at 0, 3, 5, 7, 14, 21, and 28 dpi, respectively. **(a2–f2)** The interactive 3D surface plots of the selected rectangular area in **(a1–f1)** respectively; pixels with positive signals for the two probes were indicated in blue. **(a3–f3)** Analyses of co-localization of IgM and the fpIgR according to the absorbance of the two fluorescence probes in the selected area. **(a4–f4)** Pie chart showing the percentage of IgM and fpIgR co-localization.

The co-localization between IgM and the fpIgR in the intestinal mucosa was measured by using ImageJ at different times. New merged images of **Figure 1** were generated using green and red to represent the positive signals of the fpIgR and IgM, respectively (**Figure 2**). A rectangular area in the epithelial layer was selected (**Figures 2, a1–f1**), and the interactive 3D surface plot of this area was developed; pixels with positive signals for two probes were indicated in blue, representing co-localization of IgM and the fpIgR (**Figures 2, a2–f2**). The optical density of green and red fluorescence in the selected area was measured by ImageJ, and the background color and noise were eliminated by setting a threshold automatically for each channel. The optical density was plotted and the overlapping pixels on the abscissa were considered to be the co-localized area of IgM and the fpIgR (**Figures 2, a3–f3**), and percentage values were displayed on a pie chart (**Figures 2, a4–f4**). Results indicated that 150 pixels were obtained in the selected area, of which 12% represented co-localization of IgM and the fpIgR, 24% represented single IgM and the fpIgR signals, and 40% of the selected area had no fluorescence before immunization (0 day), while the percentage values of co-localization between IgM and the fpIgR were 37% at 3 days, 58% at 7 days, 55% at 14 days, 39% at 21 days, and 35% at 28 days after immunization (**Figures 2, a4–f4**), which revealed transepithelial transport of the IgM–fpIgR (SC) complexes.

**Figure 3:**
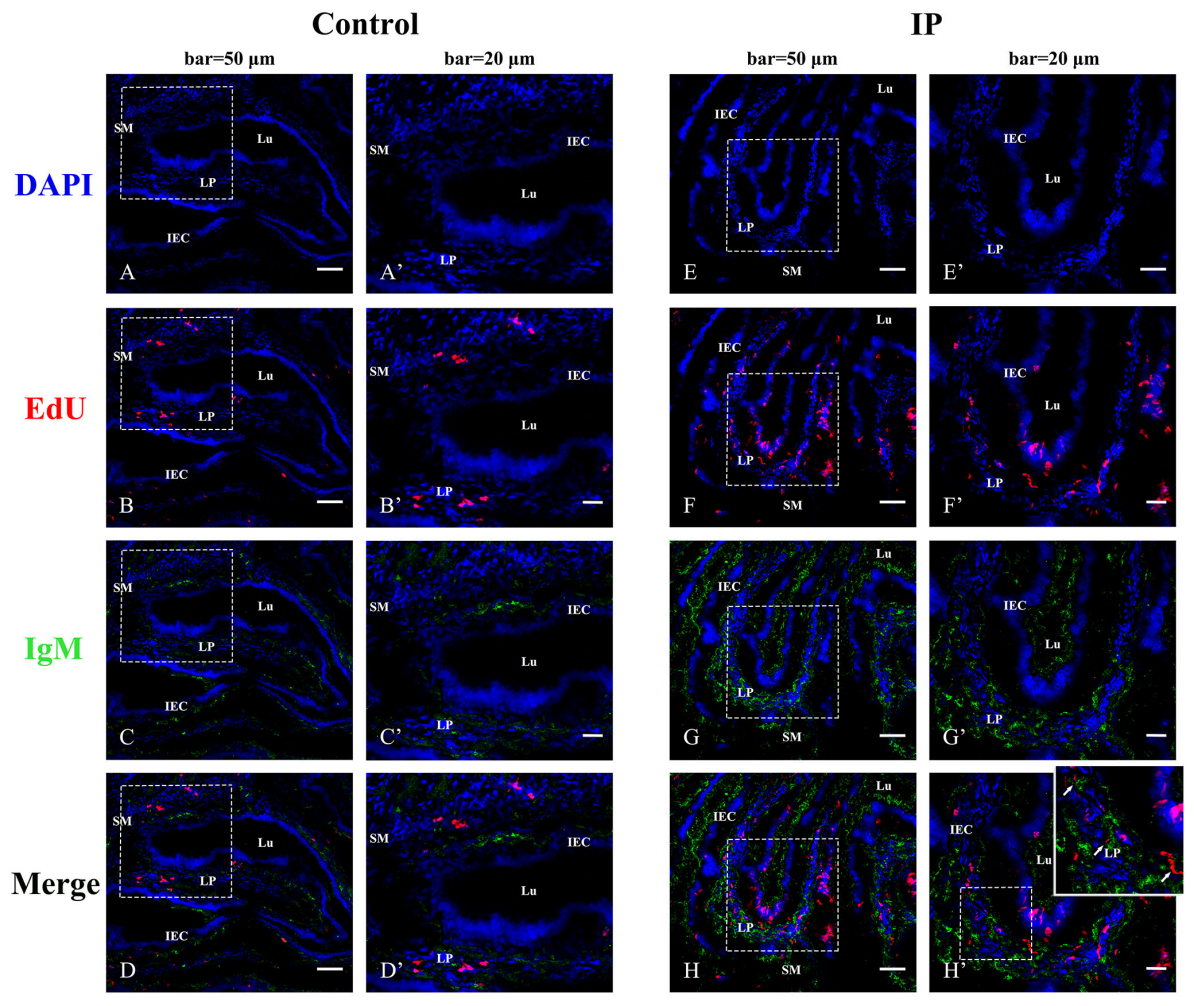
Indirect immunofluorescence for co-localization of EdU and IgM in flounder hindgut. The cell nuclei were counterstained in blue with DAPI. **(A–D)** The distribution of EdU and IgM in the PBS-control group at 48 h after EdU injection. Bar = 50 µm. **(A′–D′)** The higher magnification view of the insert area (white box) in **(A–D)**, respectively. Bar = 20 µm. **(E–H)** The distribution of EdU and IgM in the group intraperitoneally (IP) injected with EdU for 48 h after 7 days of intraperitoneal injection with inactivated *Vibrio anguillarum.* Bar = 50 µm. **(E′–H′)** The higher magnification view of the insert area (white box) in **(E–H)**, respectively. Bar = 20 µm. LP, lamina propria; Lu, lumen; IECs, intestinal epithelial cells; SM, submucosa.

For confirming the proliferation of B cells induced by immunization, EdU was injected at 7 days post-inactivated *V. anguillarium* injection, and IgM and EdU were co-stained in the hindgut after 48 h. Abundant EdU^+^ red fluorescence signals were detected in the epithelium and the lamina propria as compared with the control that showed a few positive signals in the lamina propria, indicating cell proliferation stimulated by *V. anguillarium* vaccination. On the other hand, IgM^+^ green fluorescence signals were also obviously enhanced in the epithelium and the lamina propria, and some EdU^+^IgM^+^ cells in the lamina propria identified the presence of proliferative IgM^+^ B cells (**Figure 3**).

### 3.2 Construction of pCIneoEGFP-fpIgR Recombinant Eukaryotic Plasmid

**Figure 4:**
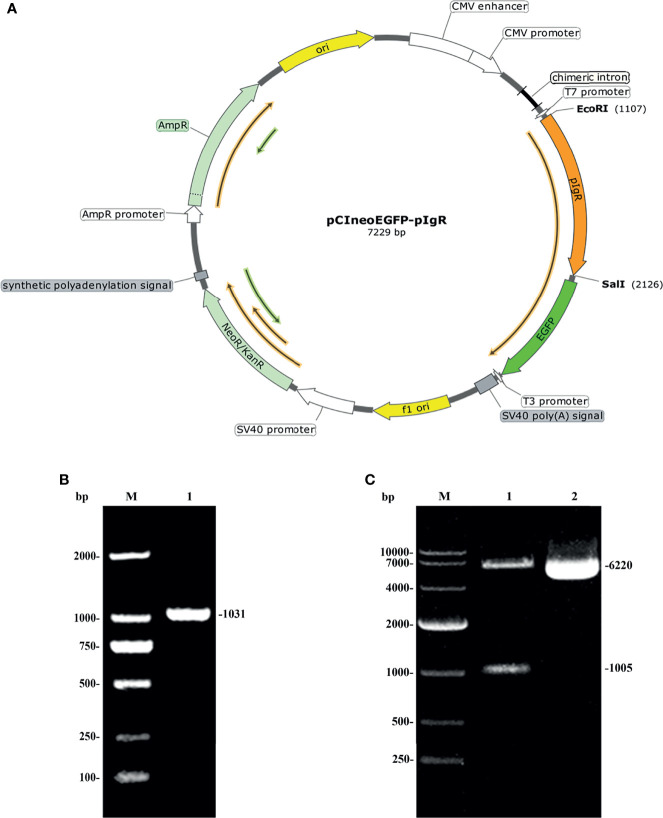
Construction of an expression vector encoding the *pIgR* of flounders. **(A)** Map of pCIneoEGFP-fpIgR eukaryotic plasmid. **(B)** Amplified fpIgR PCR products with EcoRI and SalI endonuclease sites. M: DNA marker DL 2000; lane 1: amplified PCR product. **(C)** Agarose electrophoresis of pCIneoEGFP-fpIgR eukaryotic plasmid and its double-digestion products.

The map of the recombinant eukaryotic plasmid pCIneoEGFP-fpIgR we constructed is shown in **Figure 4A**. It had a full length of 7,229 bp and contained an immediate early enhancer and promoter of cytomegalovirus, two resistance genes, ampicillin and neomycin, and a 720-bp-enhanced EGFP sequence which could be used as reporter genes for eukaryotic transfection. The *fpIgR* inserted into the multiple cloning site of the eukaryotic plasmid constituted a fusion protein with downstream EGFP and shared the same promoter and start codon. The forward primer of the specific primers used for amplification of flounders included the *Eco*RI restriction site GAATTC, the protective base CCG, and the Kozak sequence GCCACC. The reverse primer included the *Sal*I restriction site GTCGAC, the protective base GGC, and the two complementary bases TT added to prevent downstream EGFP frameshift mutations in plasmids. Identification by agarose gel electrophoresis showed that a single band of the fpIgR was amplified at 1,031 bp (**Figure 4B**).

The plasmid pCIneoEGFP and the purified fpIgR PCR product were subjected to *Eco*RI and *Sal*I endonuclease digestion and then ligated to construct the recombinant eukaryotic plasmid pCIneoEGFP-fpIgR. The positive colony was sequenced and double-digested after the plasmids had been extracted with the endotoxin-free plasmid extraction kit. Agarose electrophoresis (**Figure 4C**) showed that the digested product consisted of a 6,220-bp vector backbone and 1,005-bp *fpIgR*, indicating that the eukaryotic expression vector had been constructed. The plasmid bands were clear, without degradation, and the ratio of supercoil conformation was very high, revealing that the quality of the plasmid met the requirements for transfection.

### 3.3 Construction of the MDCK-fpIgR Cell Line

**Figure 5:**
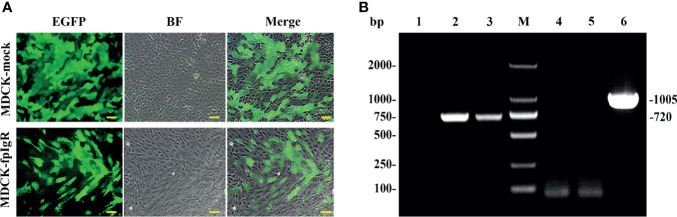
Screening of two MDCK monoclonal cell lines stably expressing EGFP and fpIgR–EGFP fusion protein. **(A)** MDCK cells stably expressed pCIneoEGFP (MDCK-mock) and pCIneoEGFP-fpIgR (MDCK-fpIgR). Bar = 50 µm. **(B)** fpIgR transcription as detected by RT-PCR. M: Marker DL 2000; lanes 1 and 4: MDCK cells; lanes 2 and 5: MDCK-mock cells; lanes 3 and 6: MDCK-fpIgR cells.

Following transfection and screening under G418 (600 μg/ml), two MDCK monoclonal cell lines stably expressing EGFP and fpIgR–EGFP fusion protein were obtained and nominated as “MDCK-mock” and “MDCK-fpIgR,” respectively. After cells had been cultured and expanded, obvious green fluorescence was observed under an inverted fluorescence microscope with uniform luminescence (**Figure 5A**), indicating the high purity of the cell line. Compared with MDCK-mock cells, MDCK-fpIgR cells had obvious morphologic changes, showing elongation and fibroblast characteristics. RT-PCR demonstrated that *EGFP* transcription with a band size of 720 bp was detected in MDCK-mock and MDCK-fpIgR cells, but the *fpIgR* of 1,005 bp was detected only in MDCK-fpIgR cells (**Figure 5B**). These two genes were absent in the untransfected negative controls.

### 3.4 Stable Expression of the fpIgR in MDCK-fpIgR Cells

**Figure 6:**
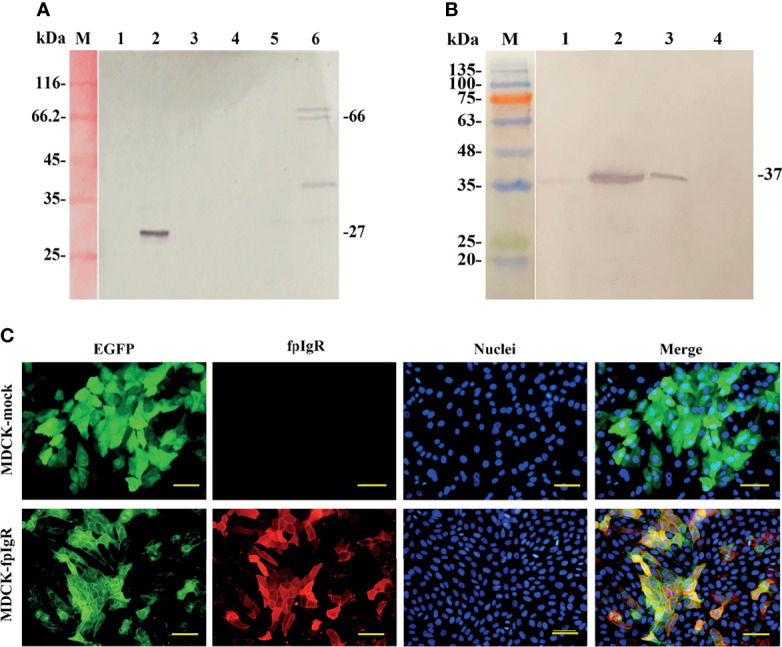
Verification of fpIgR expression in MDCK-fpIgR cells. **(A)** Western blotting of the cytosol and membrane proteins of MDCK cells. M: Molecular mass standards; lanes 1, 2, and 3: cytosol proteins of MDCK, MDCK-mock, and MDCK-fpIgR cells, respectively; lanes 4, 5, and 6: membrane proteins of MDCK, MDCK-mock, and MDCK-fpIgR cells, respectively. **(B)** Western blotting of the gut mucus of flounders and cell culture supernatants. M: Molecular mass standards; lane 1: gut mucus of flounders; lanes 2, 3, and 4: MDCK-fpIgR, flounder gill (FG) cell, and MDCK-mock cell culture supernatants without FBS, respectively. **(C)** Verification of fpIgR expression (red fluorescence signals) in MDCK-fpIgR cells by immunofluorescence staining. Bar = 50 µm.

The membrane and cytoplasmic proteins extracted from MDCK, MDCK-mock, and MDCK-fpIgR cells were subjected to Western blotting to identify fpIgR expression. The GFP tag MAb reacted mainly with the extracted cytoplasmic protein of MDCK-mock cells and membrane proteins of MDCK-fpIgR cells, forming a band of ~27 and ~66 kDa, respectively (**Figure 6A**, lanes 2 and 6), whereas no bands were present in the cytoplasmic protein of MDCK and MDCK-fpIgR cells, or the membrane proteins of MDCK and MDCK-mock cells (**Figure 6A**, lanes 1, 3–5). Western blotting revealed that mouse anti-fpIgR polyclonal antibody could recognize a single band at ~37 kDa in MDCK-fpIgR cell supernatants, showing the existence of the SC (**Figure 6B**, lane 2). Meanwhile, the anti-fpIgR polyclonal antibody could also specifically recognize an ~37-kDa protein band in the skin mucus of flounders (**Figure 6B**, lane 1) and FG cell culture supernatants (**Figure 6B**, lane 3), but not in culture supernatants of MDCK-mock cells (**Figure 6B**, lane 4). These results confirmed that the fpIgR was expressed on MDCK-fpIgR cell membranes and cleaved the extracellular region of the fpIgR as SC into cell supernatants.

Immunofluorescence staining (**Figure 6C**) demonstrated that the fpIgR showed high expression in the constructed MDCK-fpIgR cell line. fpIgR-positive red fluorescence was distributed evenly on the cell surface and co-localized with the green fluorescence of GFP. However, only green fluorescence was observed in non-fpIgR-expressing MDCK-mock cells, suggesting that distribution of the fpIgR could be reflected by the location of green fluorescence of GFP.

### 3.5 Establishment and Evaluation of Polarized Monolayers of MDCK-fpIgR Cells

**Figure 7:**
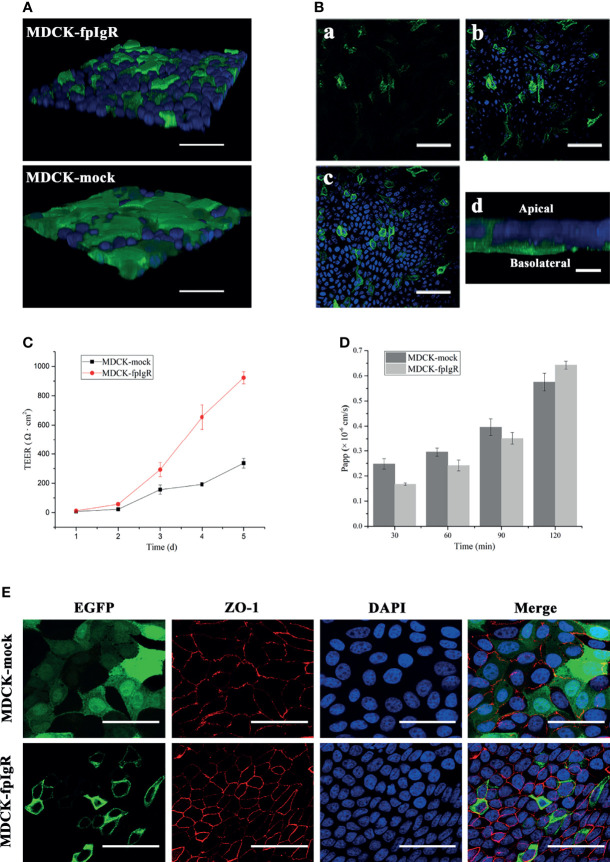
Establishment and evaluation of polarized monolayers of MDCK-fpIgR cells. **(A)** Detection of monolayers in 3D MDCK-fpIgR and MDCK-mock cells by *z*-axis CLSM after immunofluorescence staining. Bar = 50 µm. **(B)** fpIgR expression in 3D MDCK-fpIgR cells by *z*-axis CLSM after immunofluorescence staining. **(a–c)** Micrographs were representative slices of all fluorescence images. Sections from the basal portion of the cells **(a)**, the area at the middle and below the nucleus **(b)**, and at the focal plane **(c)** are shown. Bar = 50 µm. **(d)** The fpIgR was detected at the basolateral side of 3D MDCK-fpIgR cells in the *z*-axis; bar = 10 µm. **(C)** TEER results of polarized monolayer cells seeded on Transwells for 5 days. Results were the mean ± SD (*n* ≥ 3). **(D)** Apparent permeability of Lucifer yellow at different times. Results were the mean ± SD (*n* ≥ 3). **(E)** Indirect immunofluorescence of polarized monolayer cells grown on Transwells. Rabbit anti-ZO-1 polyclonal antibody served as the primary antibody. Bar = 50 µm.

MDCK-fpIgR and MDCK-mock cells were cultured on Transwell filters to form a cell monolayer for the study of the 3D cell model (**Figure 7A**). 3D MDCK-fpIgR cells were scanned by *z*-axis CLSM from the basal to the apical side of cells, and initially, fpIgR-positive green fluorescence was observed (**Figures 7B, a**), followed by the blue fluorescence of DAPI-stained nuclei (**Figures 7B, b**). Finally, some green fluorescence continued to be seen on the cell membrane when all DAPI fluorescence was observed (**Figures 7B, c**), indicating that the fpIgR was expressed mainly on the basolateral side of the cells and the cell membrane. A final z-projected image of the collected z-stack showed that most fpIgR–EGFP fusion protein was distributed on the basal side of MDCK-fpIgR cells (**Figure 7B, d**), suggesting that the monolayer of MDCK-fpIgR cells on Transwell semipermeable membrane was polarized.

MDCK-fpIgR cells were grown on 6.5-mm Transwell filters for 5 days, and TEER was measured. TEER increased with time and changed considerably from day 3 (**Figure 7C**). From day 4, TEER (192.50 ± 12.49 Ω·cm^2^) of MDCK-mock cells was ≥180 Ω·cm^2^, indicating that the cell monolayer was tightly connected and could be used for subsequent experiments. TEER of MDCK-fpIgR cells seeded at the same density was ≥180 Ω·cm^2^ if cells were grown on filters to day 3, which was significantly higher than that of MDCK-mock cells. Furthermore, MCDK-fpIgR and MDCK-mock cells cultured on Transwell filters formed a monolayer for 4 days, and the Papp of Lucifer yellow at different times in each chamber was <1.5 × 10^−6^ cm/s (**Figure 7D**), indicating that the cell monolayer on the membrane was intact, tightly connected, and not leaking.

To further verify the tightness and integrity of the cell monolayer, immunofluorescence staining of ZO-1 was undertaken when MDCK-mock and MDCK-fpIgR cell monolayers were grown on 6.5-mm filters to day 4. CLSM illustrated red fluorescence along the boundary between closely connected cells on the Transwell semipermeable membrane, and a “honeycomb” pattern outlined the typical multilateral “paving stone”-like appearance of epithelial cells (**Figure 7E**). These results demonstrated that MDCK-mock and MDCK-pIgR cell monolayers were integral and tight.

### 3.6 Verification of fpIgR-Mediated IgM Transcytosis in Polarized Monolayers of MDCK-fpIgR Cells

**Figure 8:**
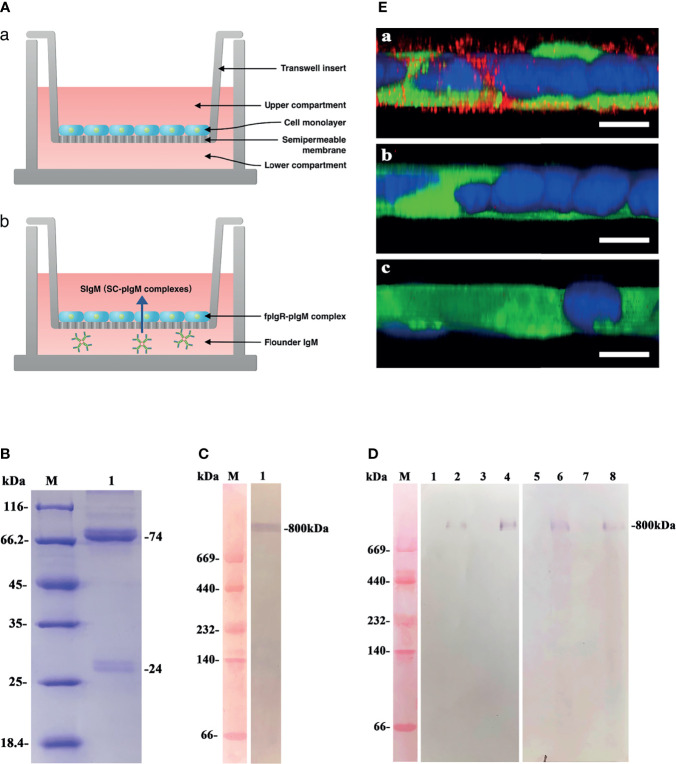
Functional evaluation of polarized MDCK-fpIgR monolayer cells to transport polymeric IgM. **(A)** Schematics of *in-vitro* Transwell assay for transcytosis of pIgM by the fpIgR. **(a)** Polarized MDCK-fpIgR cell monolayers grown on Transwell filters; **(b)** transepithelial transport of pIgM mediated by fpIgR across MDCK-fpIgR cells. **(B)** The denaturing electrophoresis result of serum IgM of flounders. Lane M: Protein molecular weight standards (kDa); lane 1: serum IgM collected. **(C)** Western blotting under native denaturing electrophoresis. Lane M: Protein molecular weight standards (kDa); lane 1: purified serum IgM at ~800 kDa. **(D)** Western blotting of IgM transported by polarized monolayer cells. Lane M: Protein molecular weight standards (kDa); lanes 1–4: samples reacted with anti-serum IgM MAb 2D8. Lanes 5–8: samples reacted with mouse anti-fpIgR polyclonal antibody. IgM transported in MDCK-mock cell lines for 1 h (lanes 1 and 5) and 2 h (lanes 3 and 7) and in MDCK-fpIgR cell lines for 1 h (lanes 2 and 6) and 2 h (lanes 4 and 8), respectively. **(E)** CLSM images of IgM transported in a 3D MDCK-fpIgR transcytosis cell model in *z*-axis. **(a)** MDCK-fpIgR cells incubated with anti-serum IgM MAb 2D8. **(b)** MDCK-fpIgR cells incubated with non-immune mouse serum. **(c)** MDCK-mock cells incubated with anti-serum IgM MAb 2D8. Bar = 50 µm.

The schematics of in-vitro Transwell assay for transcytosis of pIgM by the fpIgR we constructed are shown in **Figure 8A**. The serum IgM of flounders was purified and analyzed by SDS-PAGE under reducing conditions. Distinct protein bands at 74 and 24 kDa were observed (**Figure 8B**), corresponding to the heavy chain and light chain of IgM, respectively. Western blotting under native-PAGE conditions revealed an ~800-kDa protein band in the purified protein (**Figure 8C**), confirming that the purified protein was tetrameric IgM.

To verify that the polarized MDCK-fpIgR cell monolayers could transport polymeric IgM, purified IgM was placed into the lower compartment of Transwells, and then the cell medium in the upper compartment was collected at 1 and 2 h. Western blotting under native-PAGE conditions revealed that an ~800-kDa protein band could be recognized specifically by anti-IgM MAb 2D8 and anti-fpIgR antibody in the medium of MDCK-fpIgR cells from the upper compartment of Transwells (**Figure 8D**, lanes 2, 4, 6, 8), suggesting the existence of fpIgR (SC)–tetrameric IgM complexes. Protein bands were not found in the upper compartment of MDCK-mock cells (**Figures 8D**, lanes 1, 3, 5, 7). Upon *z*-axis CLSM of 3D MDCK-fpIgR cells, IgM-positive red fluorescence (**Figures 8E, a**) was observed in the cells after placing IgM into the lower chamber of Transwells for 1 h. In MDCK-fpIgR cells incubated with non-immune mouse serum or MDCK-mock cells, IgM-positive signals were not detected (**Figures 8E, b, c**). These results suggested that MDCK-fpIgR cells could function as a transcytosis model *in vitro*, and IgM was transported across MDCK-fpIgR cells as an fpIgR–IgM complex.

## 4 Discussion

In teleost fish, the skin-, gill-, gut-, and nose-associated lymphoid tissues have been described, and a common feature in these tissues is SIgs in the mucus (15, 29, 30). However, little is known about how the pIgR mediates SIg transport in teleosts. We previously found an fpIgR (SC)–IgM complex in the gut, skin, and gill mucus and bile after flounders had been immunized with inactivated *V. anguillarum* by intraperitoneal injection and immersion (15), and the fpIgR could mediate immune excretion of IgM–antigen complexes across the intestinal epithelium into gut mucus (23), but direct evidence for transepithelial transport of SIgM mediated by the fpIgR is lacking. In the present study, double staining of the fpIgR and IgM indicated that IgM-positive signals were mainly in the lamina propria of the gut, and fpIgR signals were mainly in intestinal epithelial cells before immunization (0 day). After flounders were immunized by immersion with inactivated *V. anguillarum*, the intensity of IgM-positive signals in the lamina propria increased from 3 dpi and then presented in intestinal epithelial cells from 7 to 28 dpi along with the fpIgR, while proliferative IgM^+^ B cells were identified in the lamina propria, suggesting that mucosal IgM antibody was locally produced in the gut mucosa. Also, quantitative analyses of images for co-localization of IgM and the fpIgR using ImageJ software demonstrated 12% of the 150 pixels in the selected rectangular area in the intestinal mucosa at the beginning (0 day), then 37% at 3 dpi, 58% at 7 dpi, 55% at 14 dpi, 39% at 21 dpi, and 35% at 28 dpi, which showed that the transepithelial transport of fpIgR–IgM complexes had occurred. Previously, we found that the levels of vaccine-specific IgM and fpIgR (SC) increased over time and that fpIgR (SC)–IgM complexes were present in the gut mucus in flounders immunized with *V. anguillarum* (15). Taken together with the data from the present study, it appeared that IgM (at least part of it) was produced in the lamina propria, and then fpIgR–IgM complexes were formed in epithelial cells and liberated to the gut mucus. However, because of the lack of fpIgR-deficient flounders, the transepithelial transport of IgM could not be verified directly to be mediated by the fpIgR *in vivo*. Hence, we developed a model system of a transfected MDCK cell line stably expressing fpIgR located mainly at the cell membrane. We verified that the polarized MDCK cell line had good tightness and integrity on the Transwell semipermeable membrane and that tetrameric IgM could be transported across polarized MDCK-fpIgR cells *via* fpIgR mediation in a basolateral-to-apical fashion.

Studies on pIgR-mediated epithelial transport of SIgs in mammals have been carried out mainly in polarized MDCK cells, a commonly used epithelial model system, and considerable progress has been made in understanding mucosal immunology (31, 32). The MDCK cell line does not express endogenous pIgR. It takes only 3–4 days for MDCK cells to differentiate into columnar epithelial cells that are tightly connected by membrane fusions of tight junctions (e.g., tight-junction protein ZO-1). Besides, the TEER of MDCK cells is not only low (similar to that in the small intestine) but also suitable for simulating the epithelial absorption and transport of substances in the intestine (31, 33). In the present study, the EGFP sequence was added to the C-terminus of the fpIgR, which did not affect the normal function of the N-terminus but could locate the fpIgR immediately. The fpIgR–EGFP fusion protein was stably distributed on the membrane of MDCK-fpIgR cells. Compared with MDCK-mock cells, MDCK-fpIgR cells with stable expression of fpIgR showed morphologic changes of elongation and fibroblast characteristics. These features are consistent with one of the principal characteristics of epithelial–mesenchymal transition in MDCK cells that overexpress human pIgR (28, 32). Moreover, the fpIgR protein expressed by eukaryotic cells should be similar to the natural pIgR of flounders in terms of glycosylation sites and native conformation. In the present study, two bands appeared near the theoretical mass of 66 kDa when the fusion proteins of pIgR-EGFP in MDCK-fpIgR cell lines were detected by mouse anti-GFP tag MAb in Western blots, which might have been because of molecular maturation caused by pIgR glycosylation as shown by pulse-chase experiments in mammals (34). Concerning the 37-kDa protein recognized by mouse anti-fpIgR polyclonal antibodies in the supernatant of MDCK-fpIgR and FG cells as well as gut mucus, it was greater than the theoretical value of the flounder SC and close to the full length of the fpIgR. This phenomenon had been documented in the skin and gut mucus of flounders, in which the SC was also ~37 kDa (21, 23). Similarly, in fugu (17) and rainbow trout (13), the molecular mass of the SC is also close to the theoretical mass of the pIgR, which may be related to post-translation modification (25), but more studies are needed to clarify this hypothesis.

In this study, the tightness and integrity of monolayers of MDCK-fpIgR cells seeded on the Transwell semipermeable membrane were evaluated by TEER, the Papp of Lucifer yellow, and ZO-1 expression. The TEER value is based on ion flow through the intercellular space, and its magnitude is proportional to the tightness of cell connections. Although TEER was measured at three sites in each filter, it might not closely reflect the permeability of the entire monolayer cell membrane. Therefore, the Papp of a marker of cell transport (Lucifer yellow) was also used to characterize it. The absorbance of a sample can reflect the integrity and tightness of the cell monolayer and is more sensitive than TEER (35). ZO-1 is a high-molecular-weight protein (~225 kDa) and is an important component of the tight connection between mucosal epithelial cells and a marker of tight junctions (36). ZO-1 expression is closely related to mucosal permeability and polarity in epithelial cells (37). In the present study, the TEER of MDCK-fpIgR and MDCK-mock cells cultured for 3–4 days was >180 Ω cm^2^, the Papp of Lucifer yellow at different times was <1.5 × 10^−6^ cm/s, and red fluorescence of ZO-1 was observed along the boundary between closely connected cells on the Transwell semipermeable membranes, and all these results collectively demonstrated that the two types of cells formed tightly connected monolayers. If cultured on a Transwell filter to form a monolayer with excellent tightness, the cell membrane can be divided into apical and basolateral regions, and the pIgR is distributed mainly on the basolateral side of the cells (7, 38). *Z*-axis CLSM images showed that the fpIgR–EGFP fusion protein was distributed mainly on the basal side of MDCK-fpIgR cells, which indicated that the monolayers of MDCK-fpIgR cells on the Transwell semipermeable membrane were polarized.

In mammals, pIgR-mediated transcytosis has been studied *in vivo* (39) and *in vitro* (6, 28). In flounders, we have reported that fpIgR can mediate the excretion of pIgM–antigen complexes across intestinal epithelial cells into gut mucus (23). However, pIgR-mediated SIg transcytosis is incompletely understood, although the pIgR–IgM complex was observed in the gut, skin, and gill mucus and bile in flounder (15). Also, other functions of the pIgR in mammals, such as intracellular neutralization (40), bacterial agglutination (41), and reduced inflammation (42), have not been clarified in teleost fish. Hence, the research model for fpIgR we established here will contribute to studies on pIgR function and mucosal immunity in fish. In this study, according to Western blotting under native-PAGE conditions, ~800 kDa proteins were IgM- and fpIgR-positive in the upper chamber of Transwells, and IgM-positive fluorescence was observed in MDCK-fpIgR cells, revealing that IgM–fpIgR (SC) complexes were secreted into cell supernatants. Taken together, these results demonstrated that the fpIgR could transport IgM molecules of flounders as tetrameric IgM–fpIgR (SC) complexes across polarized MDCK-fpIgR cells from the basolateral surface to the apical side *via* transcytosis, which was similar to that of pIgA–pIgR complexes in mammals (28, 33), and this finding improved the understanding of the mechanism underlying teleost Ig responses at mucosal sites. Unlike mammals whose pIgR requires the J-chain to bind and recognize polymeric IgA and IgM (43), the fish genome does not encode a J-chain (12, 25), but the function of the pIgR in fish (to bind and transcytose pIgM as shown in flounder) is not affected, although more research is needed to clarify this. Recently, IgT has been reported to act as a specialized mucosal Ig in teleosts (13, 29, 44) and play an important role in pathogen control and microbiota homeostasis (30), but the transport mechanism of IgT is not known. Moreover, a pIgR-like gene of flounders has been identified, it has the same functional region and conserved sequence (DxGxYWC) as the fpIgR, and a pIgR-like protein is also present in the mucus rather than in serum (45). Therefore, the functional *in-vitro* MDCK-fpIgR cell model system that we established will serve as an excellent tool for studying pIgR-mediated secretion of IgT into the mucosal lumen as well as the function of pIgR-like proteins to explore if the pIgR is the sole transport mechanism for mucosal antibodies in fish. It seems clear that mucosal Igs in teleosts, similar to those of mammals, can defend mucosa against pathogens through neutralizing or promoting their elimination and maintaining homeostasis (30), so the mucosal IgM, which is locally produced and transported across epithelial cells into secretions by pIgR at mucosal sites, will protect the mucosal health and, therefore, fish health.

In conclusion, we provided *in-vivo* evidence for transepithelial transport of the fpIgR–IgM complexes and IgM^+^ B-cell proliferation in gut-associated lymphoid tissue after immunization of flounders. To demonstrate that transepithelial transport of IgM was mediated by the fpIgR, polarized monolayers of MDCK-fpIgR cells were established because fpIgR-deficient flounders do not exist. MDCK-fpIgR cell lines with stable fpIgR expression were screened by constructing the fpIgR recombinant eukaryotic expression plasmid pCI-neo-EGFP-fpIgR and verified by RT-PCR, Western blotting, and indirect immunofluorescence. The tightness and integrity of the cell monolayer seeded on the Transwell semipermeable membranes were evaluated by TEER, the Papp of Lucifer yellow, and ZO-1 expression. Our model showed the transport of IgM from flounder from the basolateral surface to the apical side as tetrameric IgM–fpIgR (SC) complexes. Hence, polymeric IgM could pass across polarized monolayers of MDCK-fpIgR cells *via* transcytosis by the fpIgR. These new findings provide a better understanding of the cellular and molecular pathways shaping mucosal IgM responses and the mucosal immune mechanisms by which IgM functions in teleosts.

## Data Availability Statement

The original contributions presented in the study are included in the article, further inquiries can be directed to the corresponding author.

## Ethics Statement

The animal study was reviewed and approved by the Animal Care and Use Committee of the Ocean University of China.

## Author Contributions

XS designed the research, analyzed the data, and wrote the manuscript. YG, HZ, and BC performed the experiments and helped with the data analysis and manuscript writing. XT helped with the experiments and image analysis. JX and HC helped with the reagent preparation and participated in the data analysis. WZ designed the research and revised the manuscript. All authors contributed to the article and approved the submitted version.

## Funding

This study was supported by grants from the National Natural Science Foundation of China (31730101 and 31472295) and Taishan Scholar Program of Shandong Province.

## Conflict of Interest

The authors declare that the research was conducted in the absence of any commercial or financial relationships that could be construed as a potential conflict of interest.

## Publisher’s Note

All claims expressed in this article are solely those of the authors and do not necessarily represent those of their affiliated organizations, or those of the publisher, the editors and the reviewers. Any product that may be evaluated in this article, or claim that may be made by its manufacturer, is not guaranteed or endorsed by the publisher.
